# Supplementary material for: Role of an ancient light-harvesting protein of PSI in light absorption and photoprotection
Source: Nat Commun. 2021 Jan 29;12:679. doi: 10.1038/s41467-021-20967-1 (PMC7846763; doi:10.1038/s41467-021-20967-1)
Supplement: Supplementary file 1 — Supplementary Information [file 41467_2021_20967_MOESM1_ESM.pdf]

Role in light absorption and photoprotection of an ancient light-harvesting protein of PSI

Yandu Lu[a,b,*], Qinhua Gan[a], Masakazu Iwai[b,c], Alessandro Alboresi[d], Adrien Burlacot[e], Oliver Dautermann[b], Hiroko Takahashi[f], Thien Crisanto[b], Gilles Peltier[e], Tomas Morosinotto[d], Anastasios Melis[b], Krishna K. Niyogi[b,c,*]

[a]State Key Laboratory of Marine Resource Utilization in South China Sea, College of Oceanology, Hainan University, Haikou, 570228, Hainan, China

[b]Howard Hughes Medical Institute, Department of Plant and Microbial Biology, University of California, Berkeley, 94720-3102, California, USA

[c]Molecular Biophysics and Integrated Bioimaging Division, Lawrence Berkeley National Laboratory, Berkeley 94720, California, USA.

[d]Dipartimento di Biologia, Universita` di Padova, Via U. Bassi 58/B, 35121 Padua, Italy

[e]CEA, CNRS, Aix-Marseille Université, Institut de Biosciences et Biotechnologies Aix-Marseille, UMR 7265, Laboratoire de Bioénergétique et Biotechnologie des Bactéries et Microalgues, CEA Cadarache, Saint-Paul-lezDurance, F-13108 France

[f]Department of Biochemistry and Molecular Biology, Graduate school of Science and Engineering, Saitama University, Japan

*Corresponding author at:
State Key Laboratory of Marine Resource Utilization in South China Sea, College of Oceanology, Hainan University, Haikou 570228, Hainan, China.
E-mail address: ydlu@hainanu.edu.cn
Howard Hughes Medical Institute, Department of Plant and Microbial Biology, University of California, Berkeley, 94720-3102, California, USA

E-mail address: niyogi@berkeley.edu

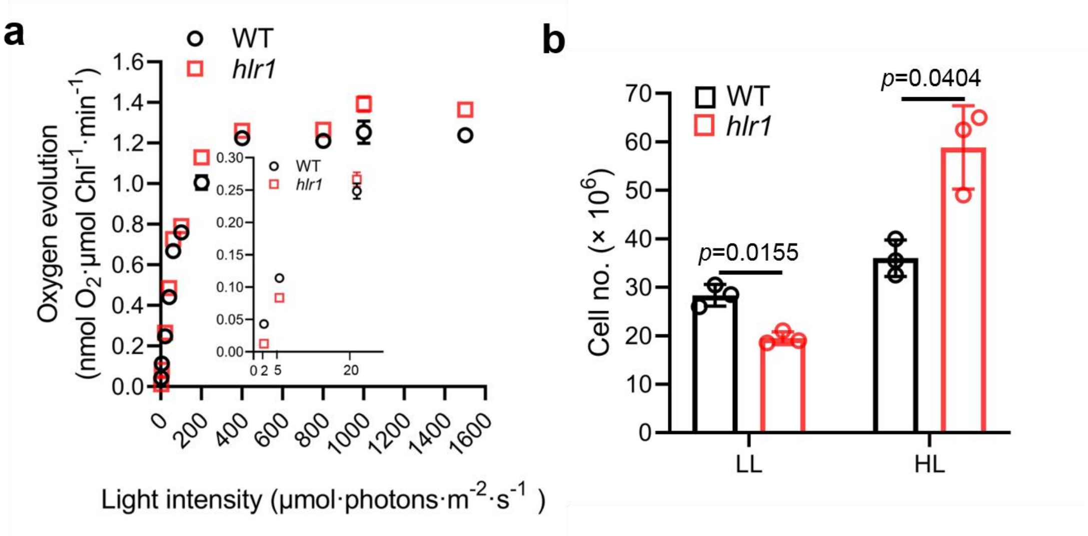

Supplementary Fig. 1. **Trade-offs between photoprotection and photosynthetic light absorption efficiency**. (**a**) The light-saturation curves of photosynthesis of WT and the *hlr1* cells acclimated to the low light conditions. The inset is a comparison of oxygen evolution rate between WT and the *hlr1* cells in the low light conditions (2, 5, and 20 μmol·photons·m^{-2}·s^{-1}). (**b**) Proliferation of WT and *hlr1* cells cultured with air bubbling in the prolonged LL (5 μmol·photons·m^{-2}·s^{-1}) or HL conditions (200 μmol·photons·m^{-2}·s^{-1}) for 8 days (the inoculation concentration is 4×10^{6}). Data are presented as the means ± SD (n = 3 for a-b). The *p* values with significance are shown.

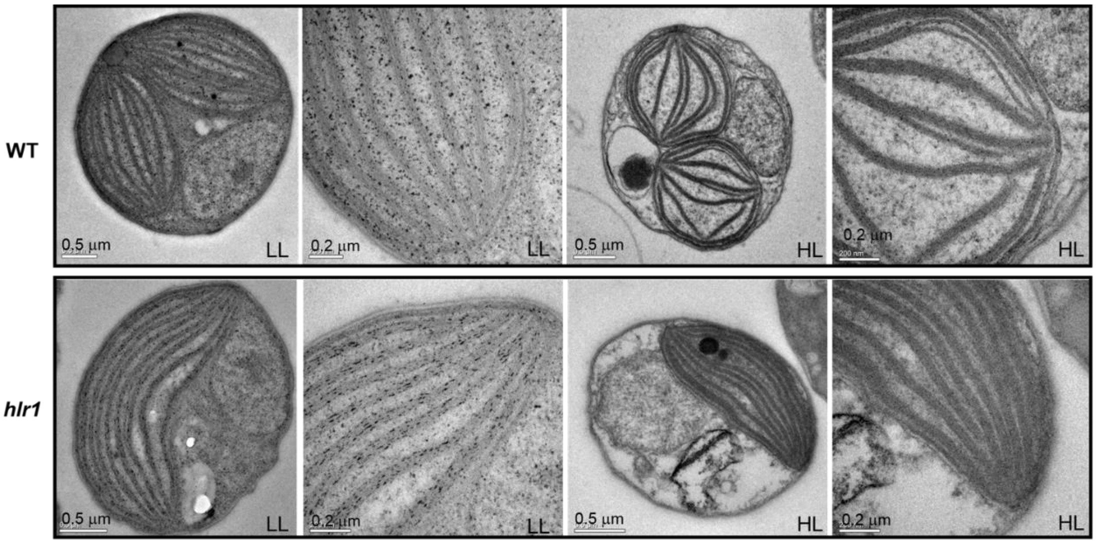

Supplementary Fig. 2. **Representative transmission electron microscopy images of WT and the *hlr1* mutant grown under the LL or HL conditions**. Scale Bars are shown. LL, low light (5 µmol·photons·m^{-2}·s^{-1}); HL, high light (200 µmol·photons·m^{-2} s^{-1}).

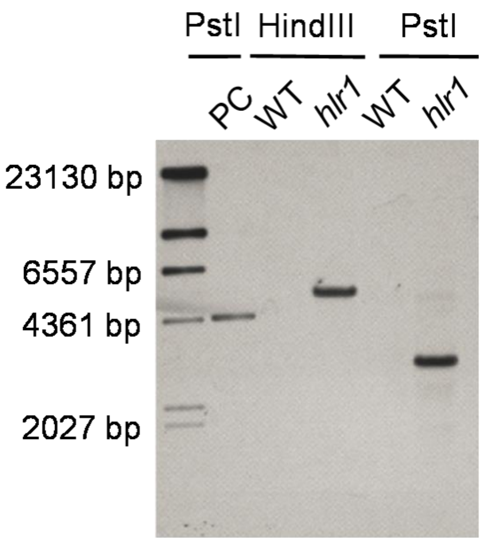

Supplementary Fig. 3. DNA gel blot analysis of the pMEM2 cassette insertion in *N. oceanica hlr1* chromosome. Genomic DNA was digested with *Pst*I or *Hind*III and probed by eHYG fragment. PC, positive controls (**pMEM2** plasmid); WT, the wild type.

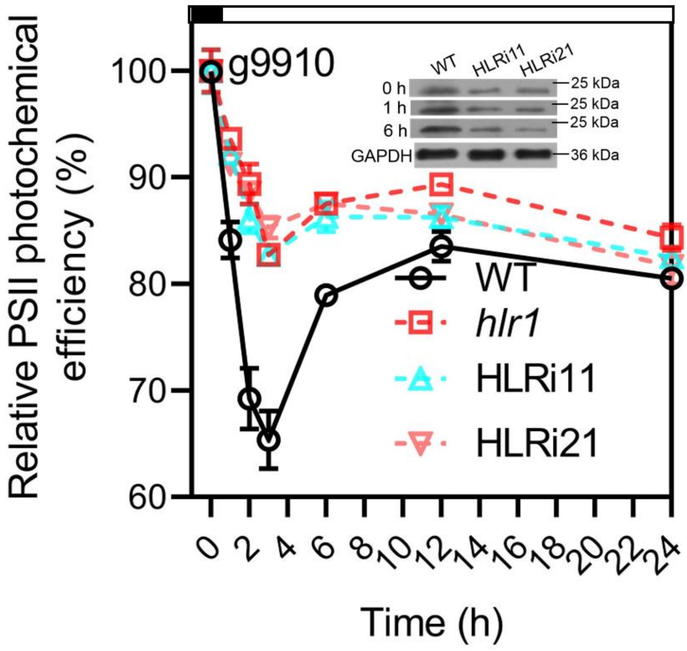

Supplementary Fig. 4. **Time course of PSII photochemical efficiency in wild type (WT), *hlr1*, and RNAi lines**. Cells were dark-acclimated overnight (black bar) followed by illumination of 200 μmol·photons·m^{-2}·s^{-1} (white bar). Inset, immunoblot analysis of HLR1 protein expression in WT and the HLR1 RNAi lines (HLRi11 and HLRi21). Protein samples of these algal cells were taken from dark-acclimated cells or following high irradiance for 6 h. Antibodies against glyceraldehyde 3-phosphate dehydrogenase (GAPDH) were used as a loading control. Data are presented as the means ± SD (n = 3).

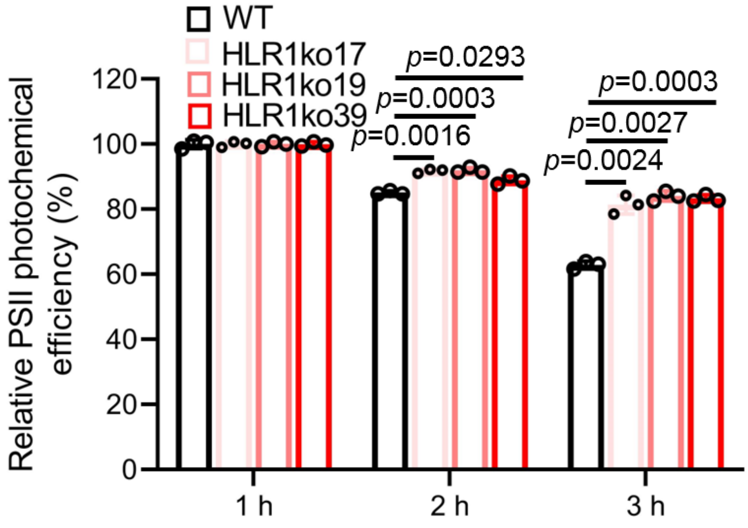

Supplementary Fig. 5. **Relative PSII photochemical efficiency as a function of time in high light of *N. oceanica* CCMP1779 wild type (WT) and HLR1 CRISPR-Cas9 knockout lines**. Cells were dark-acclimated followed by exposure to 200 μmol·photons·m^{-2}·s^{-1} for the indicated period of time. The HLR1 CRISPR-Cas9 knockout mutants (HLR1ko17, HLR1ko19, and HLR1ko39) were validated by gene cloning and sequencing. Data are presented as the means ± SD (n = 3). The *p* values with significance are shown.

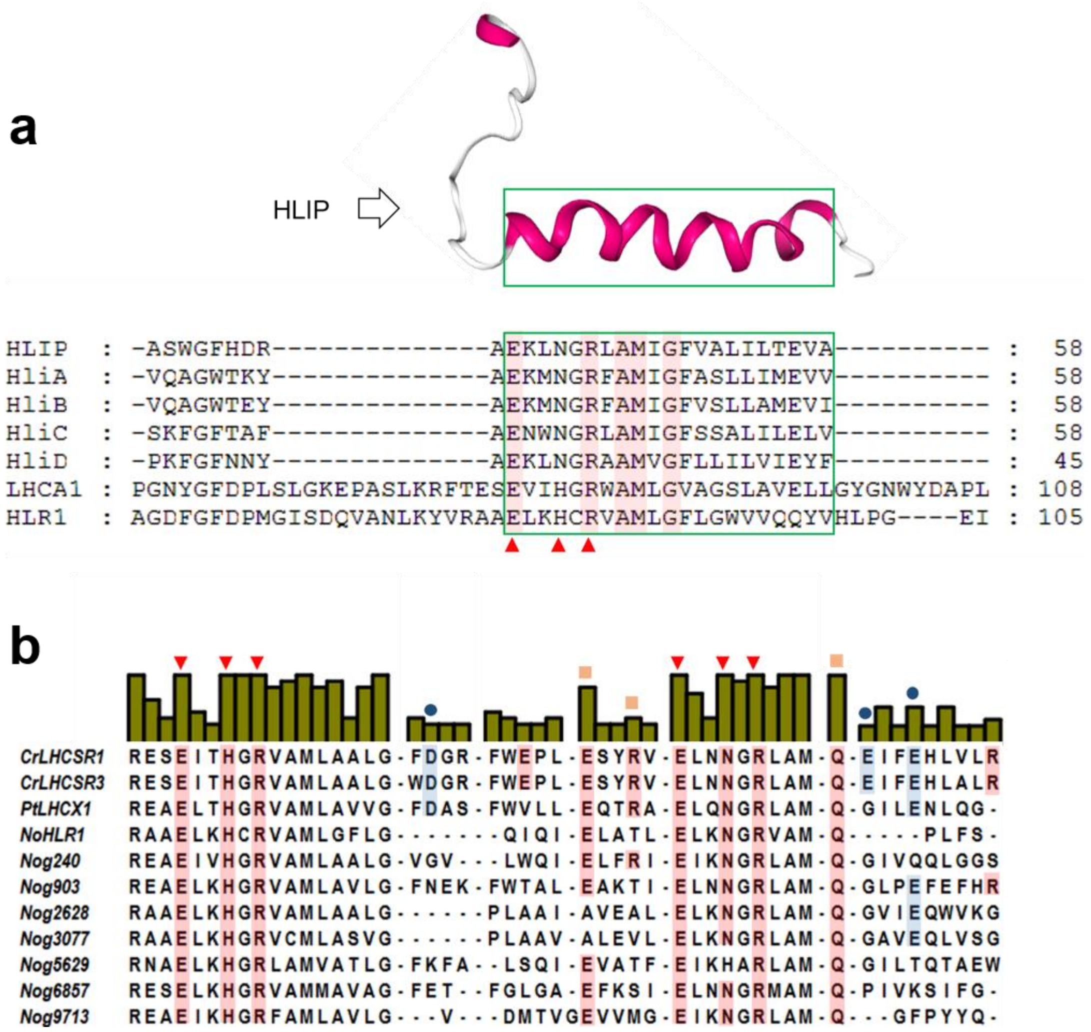

Supplementary Fig. 6. **Multiple sequence alignment of HLR1 with HLIPs or LHCSR proteins**. (a) Multiple sequence alignment of HLR1 with HLIPs. Chl *a*–specific sites are denoted by triangles. Conserved residues are highlighted in red. The folding model of an HLIP of *Synechococcus* sp. strain PCC 7942 is shown. Green box indicates the transmembrane helix in HLIPs and the TM1 in HLR1 or LHCA1. (b) Multiple sequence alignment of HLR1 with LHCSR proteins. Chl *a*–specific sites, Chl *a* and Chl *b* promiscuous sites, and the pH-sensing sites are denoted by triangles, squares, and spheres, respectively. Abbreviations: HLIP, *Synechococcus* sp. strain PCC 7942 HLIP (AAC43401.1); HliA – D, HLIPs of *Synechocystis* sp. Strain PCC6803 (ssl2542, ssr2595, ssl1633, and ssr1789); LHCA1, Light-harvesting chlorophyll *a*/*b*-binding protein of photosystem I of *C. reinhardtii* (Q05093); CrLHCSR1 and

CrLHCSR3, stress-related chlorophyll *a*/*b*-binding protein 1 and 3 of *C. reinhardtii*; PtLHCX1, *P. tricornutum* LHCX1 (protein ID 27278).

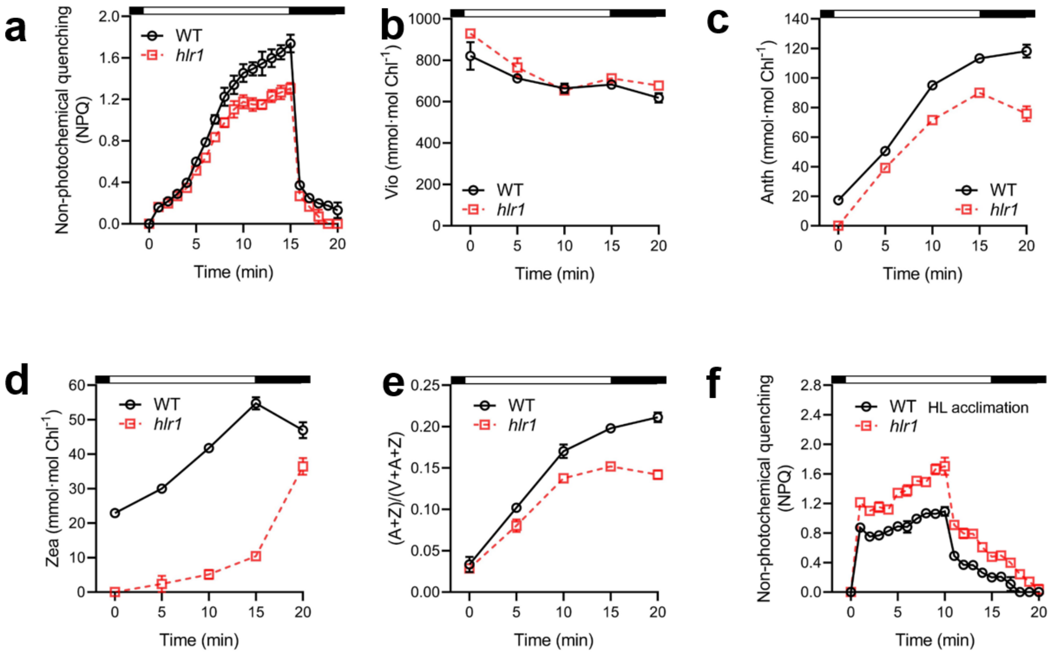

Supplementary Fig. 7. **NPQ properties of wild type (WT) and the *hlr1* mutant**. (**a**) NPQ values in response to dark/high-light exposure of WT (black circles) and *hlr1* mutant (red squares) acclimated in darkness overnight. Bars at the top of the figures indicate the time sequence of actinic light on (white bar, 200 μmol·photons·m^{-2}·s^{-1}) and off (black bars). (**b**) Kinetics of violaxanthin (Vio) accumulation determined by time-resolved HPLC measurements. (**c**) Kinetics of antheraxanthin (Anth) accumulation determined by time-resolved HPLC measurements. (**d**) Comparison of altered zeaxanthin (Zea) accumulation of WT and the *hlr1* mutant in response to high-light exposure. (**e**) Comparison of (A+Z)/(V+ A+Z) values of WT and the *hlr1* mutant. V, violaxanthin; A, antheraxanthin; Z, zeaxanthin. (**f**) NPQ values in response to dark/high-light exposure of WT (black circles) and *hlr1* mutant (red squares) acclimated in 200 μmol·photons·m^{-2}·s^{-1} for 24 h. Data are presented as the means ± SD (n = 5 for a; n = 3 for b, c, e, and f; n = 4 for d).

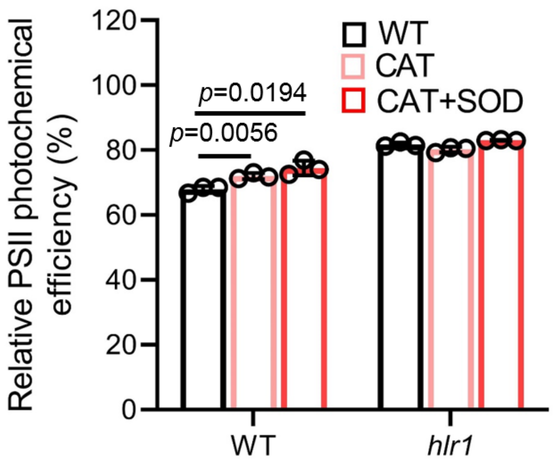

Supplementary Fig. 8. Effects of catalase (CAT) and superoxide dismutase (SOD) on HL-induced photoinhibition in WT and the *hlr1* mutant. Data are presented as the means ± SD (n = 3). The *p* values with significance are shown.

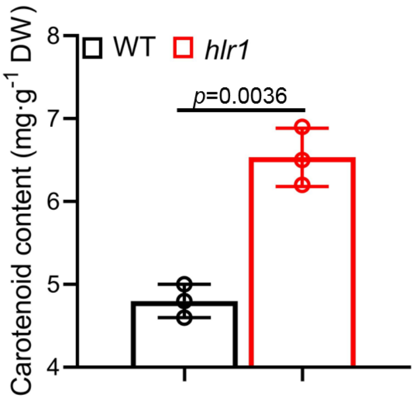

Supplementary Fig. 9. Carotenoid content of *N. oceanica* WT and *hlr1* cells cultured under HL conditions for 8 days (the inoculation concentration is 4×10^6). Data are presented as the means ± SD (n = 3). The *p* value is shown.

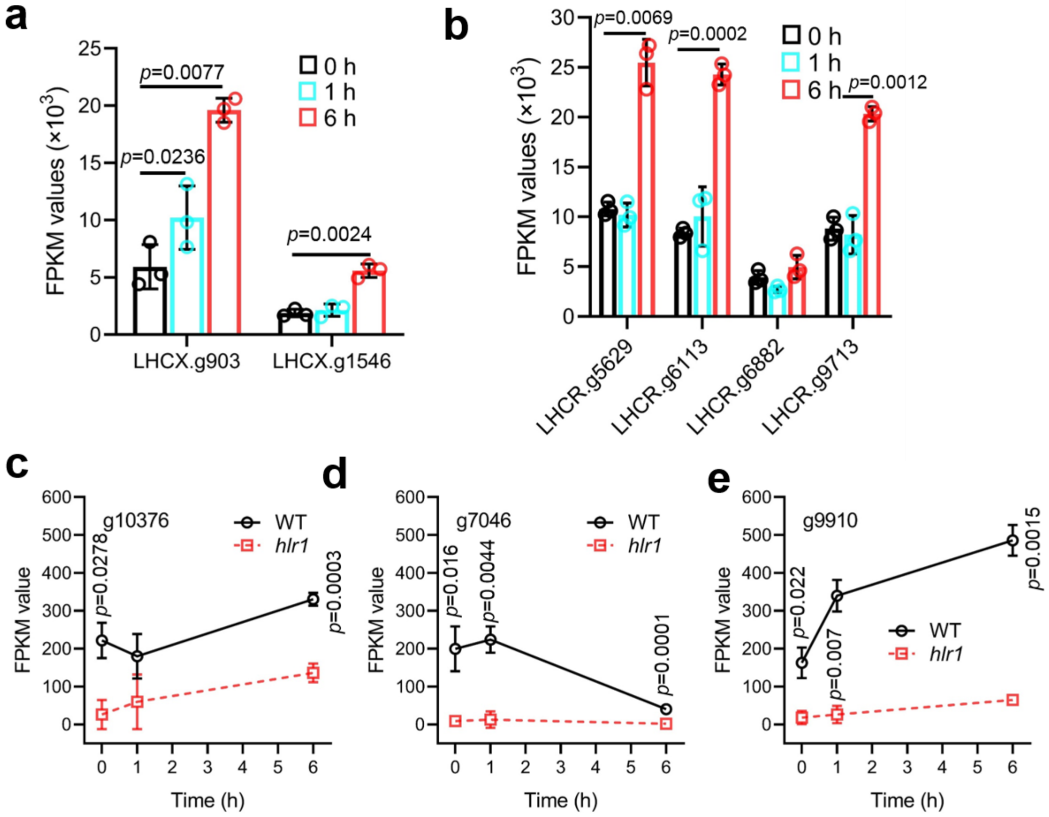

Supplementary Fig. 10. Transcript dynamics of *N. oceanica* wild type and *hlr1* cells used for RNA-Seq analysis. (a - b) Transcriptional dynamics of the genes encoding LHCXs (a) and LHCRs (b) in wild type *N. oceanica* in response to HL (200 μmol·photons·m^{-2}·s^{-1}). (c - e) Transcriptional dynamics of genes involved in ascorbic acid biosynthesis (*g10376*; c), cell wall biosynthesis (*g7046*; d), and protein glycosylation (*g9910*; e) in *N. oceanica* wild type and *hlr1* cells in response to HL (200 μmol·photons·m^{-2}·s^{-1}). Data are presented as the means ± SD (n = 3). The *p* values with significance are shown.
