# Supplementary material for: Role of an ancient light-harvesting protein of PSI in light absorption and photoprotection
Source: Nat Commun. 2021 Jan 29;12:679. doi: 10.1038/s41467-021-20967-1 (PMC7846763; doi:10.1038/s41467-021-20967-1)
Supplement: Supplementary file 3 — Descriptions of Additional Supplementary Files [file 41467_2021_20967_MOESM3_ESM.pdf]

Descriptions of Additional Supplementary Files

Supplementary data 1

Description: Molecular investigation of the hlr1 mutant. Flanking sequence of eHYG-DNA cassette in the hlr1 chromosome. The nucleotides in pink represent Nannochloropsis genomic sequence and the ones blue-highlighted are the reverse complemented sequences of the transforming cassette of vector pMEM02.

Supplementary data 2

Description: Detection of the insertion of eHYG-DNA cassette in the hlr1 mutant by transcriptomics investigation. Transcriptomics datasets of the hlr1 mutant under darkness and high irradiance were collected and assembled (15 high-quality transcript profiles over three time points, 0 h, 1 h, 6 h, 96 h, 144 h; for details, see Methods). The transforming cassette harboring the eHYG gene was identified only in scaffold c32798_g14 of the transcriptome assembly.

Supplementary data 3

Description: List of proteins identified by mass spectrometry in the BN-PAGE bands. Unused, the protein pilot score (the results are considered significant if the score values are ≥1.3); % Cov (95), the coverage of the peptides with a credibility ≥95%; Peptides(95%), the numbers of peptides with a coverage ≥95% (the results are reliable if the numbers are ≥2).

Supplementary data 4

Description: Transcriptional comparison of the transcripts of the high-light- (HL) and dark-grown (DK) WT and the hlr1 mutant cells.

Supplementary data 5

Description: The primer sequences used in this study.

Supplementary data 6

Description: Full protein list used for the phylogenetic tree of light-harvesting complex (LHC) proteins. LHCA: Chlamydomonas reinhardtii (Chl rei), Volvox carteri (Vol car), Gonium pectorale (Gon pec), Tetradesmus obliquus (Tet obl), Chlorella variabilis (Chl var). HLR1 – LHCR5: Nannochloropsis gaditana (Nan gad), Nannochloropsis salina (Nan sal), Cyanidioschyzon merolae (Cya mer), Gracilariopsis chorda (Gra cho), Chondrus crispus (Cho cri), Aureococcus anophagefferens (Aur ano), Galdieria sulphuraria (Gal sul), Vitrella brassicaformis (Vit bra), Griffithsia japonica (Gri jap), Ectocarpus siliculosus (Ect sil), Phaeodactylum tricornutum (Pha tri), Thalassiosira pseudonana (Tha pse), Thalassiosira oceanica (Tha oce).

Supplementary data 7

Description: The mRNA-Seq data for the genes involved in cell wall biosynthesis, protein glycosylation, L-ascorbic acid biosynthesis and genes encoding transcriptional factors, signaling proteins, electron transport proteins, stress-related proteins, and proteins involved in cell cycle.
